# Risk factors for pulmonary complications after percutaneous nephrolithotomy: A retrospective observational analysis

**DOI:** 10.1097/MD.0000000000004513

**Published:** 2016-09-02

**Authors:** Jihion Yu, Jae Moon Choi, Joonho Lee, Koo Kwon, Yu-Gyeong Kong, Hyungseok Seo, Jai-Hyun Hwang, Hyung Keun Park, Young-Kug Kim

**Affiliations:** a Department of Anesthesiology and Pain Medicine, Asan Medical Center, University of Ulsan College of Medicine, Seoul; b Department of Anesthesiology and Pain Medicine, Dankook University Hospital, Cheonan-si; c Department of Urology, Asan Medical Center, University of Ulsan College of Medicine, Seoul, Republic of Korea.

**Keywords:** percutaneous nephrolithotomy, postoperative pulmonary complications, risk factors

## Abstract

Although percutaneous nephrolithotomy is minimally invasive, it is associated with several complications, including extravasation of fluid and urine, the need for a blood transfusion, and septicemia. However, little is known about pulmonary complications after this procedure. Therefore, we aimed to evaluate the risk factors for and outcomes of pulmonary complications after percutaneous nephrolithotomy.

All consecutive patients who underwent percutaneous nephrolithotomy between 2001 and 2014 were identified and divided into group A (no clinically significant pulmonary complications) and group B (clinically significant pulmonary complications). Preoperative and intraoperative variables and postoperative outcomes were evaluated. Independent risk factors for postoperative pulmonary complications were evaluated by univariate and multivariate logistic regression analyses.

The study included 560 patients: 378 (67.5%) in group A and 182 (32.5%) in group B. Multivariate logistic regression analysis revealed that the independent risk factors for pulmonary complications after percutaneous nephrolithotomy were a higher body mass index (odds ratio = 1.062, *P* = 0.026), intraoperative red blood cell transfusion (odds ratio = 2.984, *P* = 0.012), and an intercostal surgical approach (odds ratio = 3.046, *P* < 0.001). Furthermore, the duration of hospital stay was significantly longer (8.4 ± 4.3 days vs 7.6 ± 3.4 days, *P* = 0.010) and the intensive care unit admission rate was significantly higher [13 (7.1%) vs 1 (0.3%), *P* < 0.001] in group B than in group A.

Risk factors for pulmonary complications after percutaneous nephrolithotomy were a higher body mass index, intraoperative red blood cell transfusion, and an intercostal surgical approach. Postoperative pulmonary complications were associated with poor outcomes. These results may provide useful information for the perioperative management of pulmonary complications after percutaneous nephrolithotomy.

## 1 Introduction

Percutaneous nephrolithotomy was first reported by Fernström and Johansson ^[1]^ in 1976. It is now recognized as an essential procedure for the removal of large, multiple, and complex renal stones.^[2]^ The procedure has progressed remarkably with the practical application of balloon dilatation in the tract, utilization of flexible nephroscopy, development of intracorporeal lithotripters, and placement of a thinner nephrostomy tube. Thus, percutaneous nephrolithotomy has achieved a higher stone-free rate and shorter post-treatment period.^[3]^ Nevertheless, percutaneous nephrolithotomy can induce severe adverse events ≥ grade 3 according to the modified Clavien classification.^[4]^

One postoperative complication that is of concern is a pulmonary complication. Postoperative pulmonary complications have a higher incidence than and an equal importance to postoperative cardiovascular complications, and are associated with high morbidity and mortality after noncardiac surgeries.^[5–7]^ Percutaneous nephrolithotomy has an increased risk for postoperative pulmonary complications because the procedure is performed near the diaphragm. Especially when approached through the upper pole of the kidney, the pleural cavity and lung could be injured. Moreover, the usage of a large volume of irrigation fluid could cause pulmonary congestion and edema.^[8]^ Meticulous surgical and anesthetic management is therefore required to reduce postoperative pulmonary complications and improve perioperative outcomes, in particular, the assessment of risk factors for postoperative pulmonary complications to predict and prevent perioperative complications. However, there is little information about pulmonary complications after percutaneous nephrolithotomy.

In the present study, we evaluated the risk factors for pulmonary complications after percutaneous nephrolithotomy. We also evaluated postoperative outcomes such as the duration of hospital stay and intensive care unit admission rate.

## 2 Methods

### 2.1 Study population

The study protocol was approved by the Asan Medical Center Institutional Review Board (approval number: 2014-0975). A retrospective review of the computerized electronic medical records of our hospital was performed for patients who underwent percutaneous nephrolithotomy for renal stones between January 2001 and August 2014. Patients were excluded if they had repeat percutaneous nephrolithotomy, no postoperative chest X-ray, or incomplete medical records.

### 2.2 Anesthetic technique

Anesthesia was performed according to the standardized clinical protocol of our institution. General anesthesia was induced with thiopental. Anesthesia was maintained with sevoflurane or desflurane, a 50% O_2_-air mixture, and fentanyl. Vecuronium or rocuronium was used as a muscle relaxant. Fluids such as crystalloid (lactated Ringer's solution) and colloid (6% hydroxyethyl starch) were administered during surgery. Arterial blood pressure was continuously monitored using a radial artery catheter. Mean arterial blood pressure was maintained above 65 mm Hg by fluid administration and vasoactive drugs such as phenylephrine and ephedrine. Packed red blood cells were transfused when the hemoglobin concentration was <8 g/dL.

### 2.3 Percutaneous nephrolithotomy

The surgical procedure was performed as follows: first, a ureteral catheter was inserted by cystoscopy with the patient in a lithotomy position; then, the patient was moved to a prone position. After the position of the ureteral catheter was confirmed by fluoroscopy, a puncture was made using an intercostal or subcostal approach. A guide wire was inserted, and the track was dilated serially by balloon dilatation. After a 34-Fr working sheath was inserted, stones were fragmented by an ultrasonic lithotripter, a ballistic lithotripter, or a holmium:YAG laser. A grasp was used to remove the fragmented stones, and a 14-Fr Malecot nephrostomy tube was placed in the renal pelvis to drain urine. Immediately after percutaneous nephrolithotomy, an arterial blood gas analysis, chest X-ray, and laboratory tests were performed.

### 2.4 Data collection

We included patients who underwent percutaneous nephrolithotomy during the study period and collected their demographic data, laboratory values, intraoperative data, and postoperative outcomes. Information on preoperative variables such as age, gender, body mass index, comorbidities (hypertension, diabetes mellitus, and respiratory, cardiovascular, and cerebrovascular disease), smoking history, operation history, and hematocrit, albumin, and creatinine levels were also collected. Hypertension was defined as a systolic arterial blood pressure > 140 mm Hg and diastolic arterial blood pressure > 90 mm Hg, or the use of anti-hypertensive drugs. Respiratory disease included chronic obstructive pulmonary disease, pulmonary tuberculosis, and asthma, regardless of medical treatment. Cardiovascular disease included coronary artery disease and heart failure. Cerebrovascular disease included having had a carotid artery stent or carotid artery angioplasty, and a history of transient ischemic attack, stroke, and cerebral hemorrhage. Smoking history was categorized into active or nonsmoker. Active smoker was defined as smoking at least once in the year before surgery. Operation history included patients who had undergone a laparotomy or cardiothoracic operation.

Information on intraoperative variables such as duration of anesthesia, fluid administration, red blood cell transfusion, stone characteristics (number, location, largest size, and type), and surgical technique were also collected. Stone number was categorized into single or multiple. Stone location was categorized into pelvis, calyx, diverticulum, ureteropelvic junction, and multiple locations. Stone size comprised the maximum length of the largest stone viewed by kidney–ureter–bladder radiography or intravenous pyelogram. Stone type was categorized into staghorn or nonstaghorn.

Postoperative outcomes included the duration of hospital stay and intensive care unit admission rate. The duration of the hospital stay was determined starting the day after percutaneous nephrolithotomy. The intensive care unit admission rate was determined by the number of patients admitted to the intensive care unit after percutaneous nephrolithotomy.

### 2.5 Definition of postoperative pulmonary complications

Kroenke et al^[9]^ defined postoperative pulmonary complications as follows: grade 1, dry cough, microatelectasis, and dyspnea, not due to other documented cause; grade 2, productive cough, bronchospasm, hypoxemia, atelectasis, hypercarbia, and an adverse reaction to pulmonary medication; grade 3, pleural effusion, suspected and proved pneumonia, pneumothorax, and the need for reintubation; and grade 4, ventilation failure. Importantly, Hulzebos et al^[10]^ defined a clinically significant postoperative pulmonary complication as comprising of 2 or more grade 2 complications or 1 grade 3 or 4 complication. Pulmonary complications were considered subclinical when only abnormal radiological findings were found without clinical symptoms or changes in auscultation (e.g., grade 1 postoperative pulmonary complications). In the present study, the patients were divided into group A, no clinically significant postoperative pulmonary complications (i.e., the patients did not develop complications or developed grade 1 complications), and group B, clinically significant postoperative pulmonary complications (i.e., the patients with 2 or more grade 2 complications or 1 grade 3 or 4 complication).^[11]^

### 2.6 Statistical analysis

Continuous variables are expressed as mean ± SD or median (interquartile range) and were compared using a *t* test and Mann–Whitney *U* test. Categorical variables are presented as number (percentage) and were analyzed using chi-square test or Fisher's exact test. The most relevant factors associated with postoperative pulmonary complications were included in the univariate logistic regression analysis. Variables with *P* values <0.1 in the univariate logistic regression analysis were included in a stepwise multivariate logistic regression analysis to evaluate the independent risk factors for pulmonary complications after percutaneous nephrolithotomy. In all other analyses, except the univariate logistic regression analysis, a *P* value <0.05 was considered statistically significant. All analyses were performed using SPSS version 21.0 software (IBM Corp., Armonk, NY).

## 3 Results

**Figure 1:**
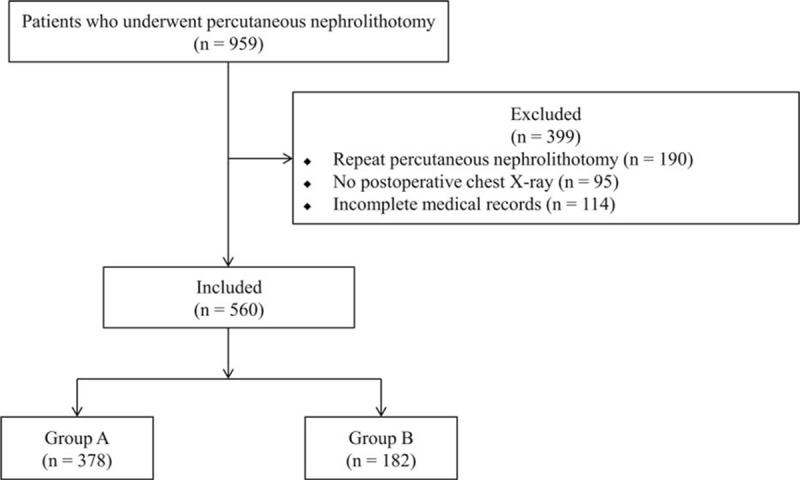
Flowchart of study participants.

Of the 959 patients who underwent percutaneous nephrolithotomy for renal stones during the study period, 399 were excluded from the study because of repeated percutaneous nephrolithotomy (n = 190), no postoperative chest X-ray (n = 95), and incomplete medical records (n = 114). The remaining 560 patients were included. Group A was comprised of 378 (67.5%) patients and group B was comprised of 182 (32.5%) (Fig. 1).

The preoperative variables in the 2 groups are summarized in Table 1. There were significant differences in age and body mass index between the 2 groups (*P* = 0.044 and 0.002, respectively; Table 1), but no significant differences in comorbidities. Table 2 shows the intraoperative variables in the 2 groups. There were significant differences in stone location and surgical approach between the 2 groups (*P* = 0.008 and *P* < 0.001, respectively).

**Table 1:**
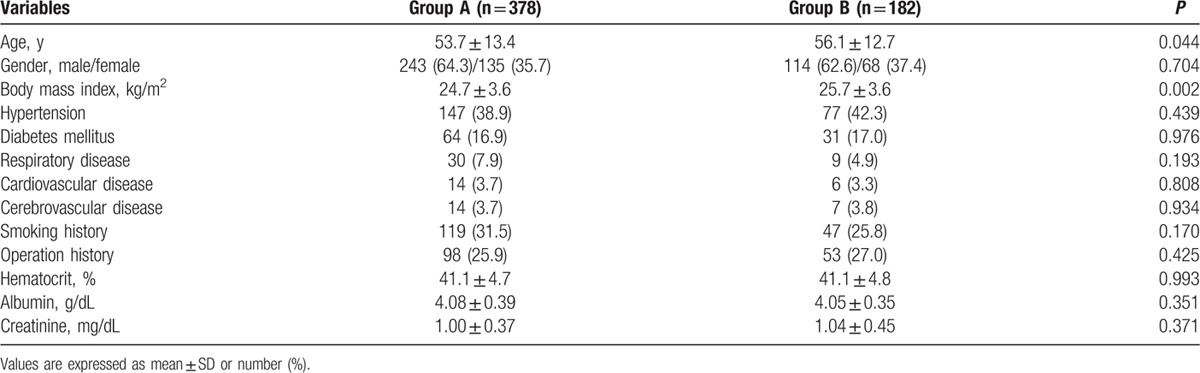
Preoperative variables.

**Table 2:**
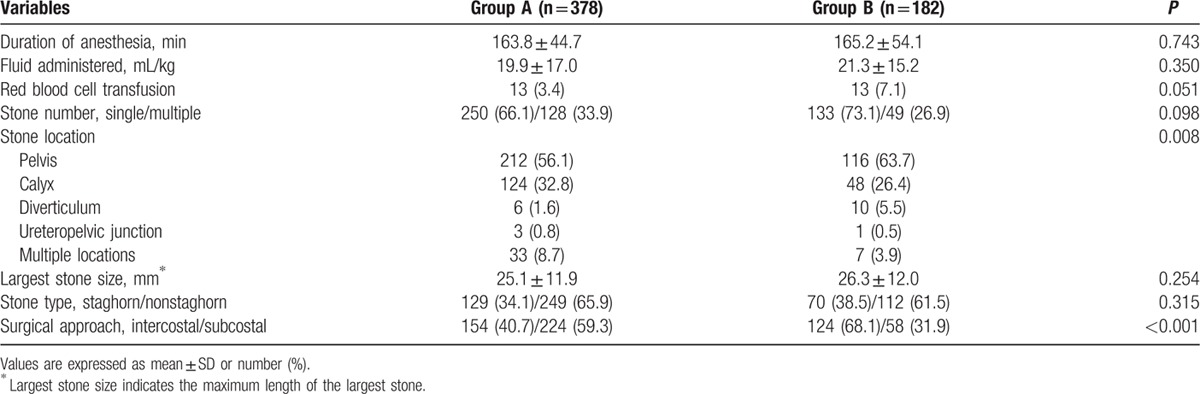
Intraoperative variables.

A univariate logistic regression analysis revealed that age, body mass index, intraoperative red blood cell transfusion, stone number, stone location, and surgical approach were associated with postoperative pulmonary complications (Table 3). A multivariate logistic regression analysis revealed that a higher body mass index (odds ratio = 1.062, *P* = 0.026), intraoperative red blood cell transfusion (odds ratio = 2.984, *P* = 0.012), and an intercostal surgical approach (odds ratio = 3.046, *P* < 0.001) were significantly associated with pulmonary complications after percutaneous nephrolithotomy (Table 3).

**Table 3:**
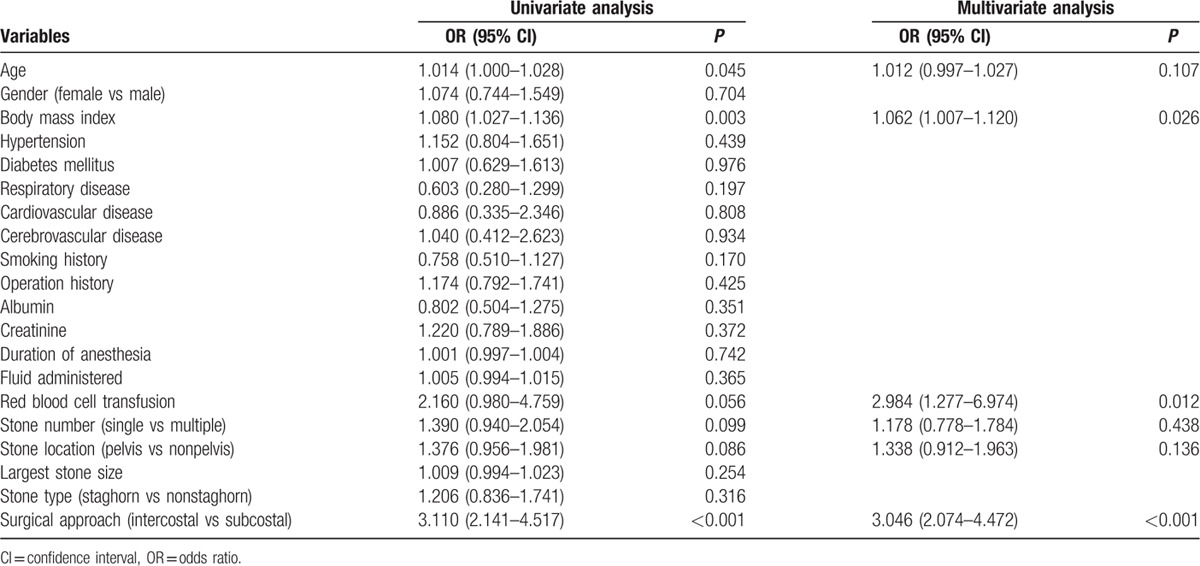
Univariate and multivariate logistic regression analyses for predictors of postoperative pulmonary complications.

The duration of hospital stay was significantly longer in group B than in group A (8.4 ± 4.3 days vs 7.6 ± 3.4 days, *P* = 0.010), and the intensive care unit admission rate was significantly higher in group B than in group A (13 [7.1%] vs 1 [0.3%], *P* < 0.001).

## 4 Discussion

In this study, the incidence of clinically significant postoperative pulmonary complications in patients who underwent percutaneous nephrolithotomy was 32.5%, which was higher than that of a previous study.^[8]^ Also, a higher body mass index, intraoperative red blood cell transfusion, and an intercostal surgical approach were associated with these complications.

Postoperative pulmonary complication rates may vary according to surgical procedures, anesthesia types, and patient conditions. Postoperative pulmonary complications are more frequent following thoracic surgery (37.8%) than following upper abdominal (12.2%) or peripheral (2.2%) surgery.^[12]^ Especially, the postoperative pulmonary complication rates after abdominal surgery were reported to range between 10% and 80%, depending on the definition of the complications.^[13]^ In our study, to appropriately reflect the clinical significance, the definition of postoperative pulmonary complications included clinical manifestations such as productive cough, atelectasis, and pleural effusion, which did not necessarily require interventions such as chest tube insertion. In addition, the characteristics of the study population such as age, body mass index, and comorbidities were dissimilar to the previous study.^[8]^ Probably due to these reasons, the incidence of postoperative pulmonary complications was higher in our study than in the previous study.^[8]^ Nonetheless, our results should be interpreted with caution because of the relatively higher incidence of postoperative pulmonary complications.

The more common perioperative complications of percutaneous nephrolithotomy were extravasation, bleeding requiring a blood transfusion, and fever; major complications, such as septicemia and colonic or pleural injury that required intervention, were very rare.^[14,15]^ In the present study, we focused on pulmonary complications after percutaneous nephrolithotomy. These were strongly associated with poor outcomes such as a prolonged stay in the intensive care unit, a prolonged period of intubation, and delayed discharge from hospital.^[16,17]^ Therefore, predicting and preventing pulmonary complications after percutaneous nephrolithotomy are important in improving postoperative outcomes.

In the present study, we found that a higher body mass index was associated with postoperative pulmonary complications. Obesity is associated with cardiovascular and respiratory diseases and increased morbidity.^[18]^ In particular, a higher body mass index increases blood loss, the need for a blood transfusion, and operating times in various urological operations.^[19,20]^ In addition, in a previous study, the hospital stay was relatively longer (4.4 vs 3.5 days) and the overall rate of complications such as bleeding, pneumonia, and pulmonary embolism was much higher (37% vs 16%) in obese than in nonobese patients after percutaneous nephrolithotomy.^[21]^ Furthermore, obesity is a risk factor for pulmonary complications after abdominal surgery.^[22,23]^ Respiratory mechanics such as lung and chest wall compliance and functional residual capacity are more impaired, and alveolar-arterial oxygenation gradients are more increased in obese than in nonobese patients.^[24]^ Therefore, meticulous pulmonary management to reduce pulmonary complications after percutaneous nephrolithotomy is especially important in obese patients.

In the present study, intraoperative red blood cell transfusion was an independent risk factor for postoperative pulmonary complications, possibly by causing systemic inflammatory response syndrome. Blood used in transfusions contains high concentrations of immune mediators, such as bactericidal/permeability-increasing protein, and triggers an inflammatory response.^[25]^ Furthermore, the leukocytes in allogeneic blood components induce immunosuppressive effects in recipients, which may negatively affect patients immunosuppressed by surgical trauma.^[26]^ Several previous studies demonstrated a relationship between a blood transfusion and pulmonary complications after cardiac and noncardiac surgery.^[6,27,28]^ Therefore, efforts to reduce the need for blood transfusion are required.^[29–31]^

We found that postoperative pulmonary complications occurred more frequently when an intercostal surgical approach was taken compared with a subcostal surgical approach. In a previous study, 15.3% of patients developed pulmonary complications after percutaneous puncture using an intercostal approach, whereas only 1.4% developed pulmonary complications after percutaneous puncture using a subcostal approach.^[32]^ The link between an intercostal approach and pulmonary complications may be anatomical. The upper pole of the kidney was located near to the posterior of the 11th to 12th rib or sometimes level with the 10th rib, and was separated from the pleural cavity by the diaphragm. Because the posterior portion of the diaphragm is attached to the inferior margin of the 12th rib, the diaphragm could be punctured if approached via the 11th to 12th intercostal space. The risk of complications is especially high when the puncture occurs above the 11th rib.^[33,34]^

In the present study, postoperative pulmonary complications were associated with a longer hospital stay and higher rate of intensive care unit admission. Previous studies also showed that postoperative pulmonary complications may be associated with a higher rate of admission to the intensive care unit and readmission to hospital, longer postoperative hospital stays, higher hospital care costs, and higher mortality in noncardiothoracic or abdominal surgery.^[7,35–37]^ Postoperative pulmonary complications resulted in severe morbidity.

This study had several possible limitations. First, the retrospective design may have meant that we did not evaluate all possible factors affecting the postoperative outcome. However, we included many important variables in our analysis to surmount this limitation. Second, a large number of patients were excluded due to insufficient medical records. However, the study had a large sample size despite this exclusion. Therefore, our results may offer useful information about predictive factors for pulmonary complications after percutaneous nephrolithotomy.

In conclusion, we found that the risk factors for pulmonary complications after percutaneous nephrolithotomy were a higher body mass index, intraoperative red blood cell transfusion, and an intercostal surgical approach. We also found that postoperative pulmonary complications were associated with a longer hospital stay and a higher rate of intensive care unit admission. Our current findings provide valuable information on perioperative management to reduce pulmonary complications and improve outcomes in patients undergoing percutaneous nephrolithotomy.
